# Moral progress: Recent developments

**DOI:** 10.1111/phc3.12769

**Published:** 2021-09-22

**Authors:** Hanno Sauer, Charlie Blunden, Cecilie Eriksen, Paul Rehren

**Affiliations:** ^1^ Department of Philosophy and Religious Studies Utrecht University Utrecht The Netherlands

## Abstract

Societies change over time. Chattel slavery and foot‐binding have been abolished, democracy has become increasingly widespread, gay rights have become established in some countries, and the animal rights movement continues to gain momentum. Do these changes count as moral progress? Is there such a thing? If so, how should we understand it? These questions have been receiving increasing attention from philosophers, psychologists, biologists, and sociologists in recent decades. This survey provides a systematic account of recent developments in the understanding of moral progress. We outline the concept of moral progress and describe the different types of moral progress identified in the literature. We review the normative criteria that have been used in judging whether various developments count as morally progressive or not. We discuss the prospects of moral progress in the face of challenges that claim that moral progress is not psychologically possible for human beings, and we explore the metaethical implications of moral progress.

## 1 INTRODUCTION

Societies change. Two hundred years ago, almost everyone in the world was poor; millions of Chinese girls' feet were bound; the US economy thrived on slavery; women were not allowed to vote; homosexuality was considered a crime; and the idea that animals should be treated humanely was met with ridicule. Nowadays, universal suffrage is without alternative, at least in most developed nations; in some countries, gay rights are firmly established; foot‐binding has been unheard of for decades; chattel slavery has long been abolished; and the animal rights movement continues to gain momentum. It would be hard to deny that many people's lives have been greatly improved. But do these improvements amount to moral progress?

For much of the 20th century, it was taken as a sign of moral progress that we had stopped believing in it (Buchanan & Powell, 2018: 4–10; Macklin, 1977: 370). However, in the 1980s and ‘90s, influential thinkers like Peter Singer (1981) and Martha Nussbaum (1993) pioneered new ways of thinking about progress that were meant to avoid the dangers of moral dogmatism and fundamentalism. Since then, the topic of moral progress has developed into a vibrant area of research, not only in philosophy, but also in fields such as psychology, biology, and sociology (though it does remain somewhat frowned upon in some corners of political theory, anthropology, and critical theory).

Along with this revitalization of moral progress came an increased interest in the topic of moral regress, with several philosophers arguing that their society's morality or circumstances had taken significant turns for the worse. For example, Bernard Williams (1985: Ch. 10) argued that our ethical life has been corrupted by the “peculiar institution” of the morality system, which collapses all of ethics into the psychologically unrealistic concept of moral obligation. According to G. E. M. Anscombe (1958), Western moral philosophy of her day was unsustainable, in part because our notions of what is morally right and wrong do not make sense in the absence of a divinely imposed moral laws, which are no longer widely accepted. Along similar lines, Alasdair MacIntyre (1981) suggested that Enlightenment morality, shorn of Aristotelean teleology, has left Western societies in a bleak state of moral incoherence.

This entry will provide a systematic summary of recent developments in the understanding of moral progress. For reasons of brevity, we will focus on the topic of moral progress, rather than moral regress. However, because moral progress and moral regress are in many ways two sides of the same coin, questions about how to understand the concept, types, criteria, prospects, and metaethical implications of moral progress may often also help to better understand moral regress. There are five sections. Sections 2 and 3 are about what moral progress consists in: Section 2 is about the concept of moral progress, Section 3 gives an overview of important types of moral progress. Section 4 is about the normative criteria used for judging various developments as progressive or not, and Section 5 reviews work on the prospects of moral progress. Finally, Section 6 discusses some of the metaethical implications of moral progress.

## 2 THE CONCEPT OF MORAL PROGRESS

Unsurprisingly, philosophers have put forward several different views of what moral progress is. The contestants can be arranged in two groups. Some propose a *Broad Conception* of moral progress: “Moral progress implies, in the first instance, a change in circumstances for the better” (Wilson, 2010: 97). Moral progress is here understood as *any kind* of morally desirable change (e.g., see Eriksen, 2020: 5–11; Kitcher, 2017: 64; Sauer, 2019). On this conception, moral progress includes a broad range of changes, from individual changes in beliefs and behavior (e.g., a father learning to be more patient with his children) over developments on a global scale (a global decline in murder rates) to improvements in moral theory.

In contrast, proponents of a *Narrow Conception* of moral progress contend that some morally desirable changes should not count as moral progress—either because these changes do not fit our intuitive understanding of moral progress, or for theoretical reasons. Thus, on this narrow view, *only some kinds* of morally desirable change should count as moral progress. One example of this is Buchanan and Powell (2018), who argue that we should only count as moral progress morally desirable changes that are brought about by “the exercise of or improvements in the moral powers” of humans (51). On this view, changes that end up benefiting humanity but happen without any human involvement (e.g., a terrible tyrant dropping dead because of cancer), should not count as instances of moral progress.

Other versions of a *Narrow Conception* result from different ways of answering the question of *what* it is that progresses when moral progress happens. For example, some contemporary theorists suggest that ultimately, moral progress happens at the level of individuals: societies progress when the moral beliefs or moral behaviours of enough of the people making up that society improve (e.g., Buchanan & Powell, 2018; Singer 1981). Others disagree, and argue that moral progress is inherently social in nature, and so should not be applied to individuals, but “only to events, institutions, and practices in countries, cultures, societies, eras, or periods in history” (Macklin, 1977: 370).

Finally, a third way to restrict the range of which morally desirable changes should count as moral progress is by narrowing the scope of moral progress. Many thinkers argue that statements about moral progress only make sense locally, for instance, about a particular person or practice in a particular society in a particular time, and not globally, such as for all humans or societies in the world (see, e.g., Hermann, 2019; Moody‐Adams, 1999: 169; Posner, 1997a, 1997b). One reason to think this is because it may be difficult or even impossible to assess the truth of global progress claims. This could be because we lack access to (some of) the relevant empirical information, or because progress in one area can lead to regress in another, making an overall assessment difficult. Moreover, any global assessment of moral progress will likely require comparing the morality of different cultures or time periods, which may turn out to be meaningless due to various issues of value incommensurability (see, e.g., Brandhorst, 2015: 230; Kitcher, 2011: 242).

## 3 TYPES OF MORAL PROGRESS

Most current discussions of different types of moral progress take place against the background of Buchanan and Powell's (2018) version of narrow moral progress (see Sect. 1). Recall that according to Buchanan and Powell's narrow conception, only improvements that came about at least in part through the exercise of moral capacities (such as moral reasoning, moral motivation, moral virtues, or the ability to follow moral norms) should count as moral progress. In contrast, broad moral progress also includes other improvements from the moral point of view.

One important type of broad moral progress involves *gains in human welfare*. While some gains to human welfare directly implicate moral capacities (such as gains in welfare due to the spread of ideas about human rights or the decline in acceptability of cruel and unusual punishments; Huemer, 2016: 1990), others need not be morally motivated. For example, starting around 1450 CE, Europe (and other places) has seen a sharp decline in the levels of violence and murder: a trend that continues to this day (Pinker, 2011). This development is no doubt welcome from a moral point of view, but it does not seem to have been caused by changes in people's moral beliefs or motivations. Buchanan and Powell (2018) suggest that this development largely came about because of the increasing ability of early modern states to maintain the peace, not because people suddenly started to become less motivated to kill each other. Similar points have been made about increasing levels of material prosperity (due largely to the proliferation of markets; Buchanan, 2020: 119–125), and higher average life expectancy (also due to markets, plus increasing scientific, medical, and technical knowledge; Pinker, 2018: chaps. 5–6; Roser et al., 2013).

What about instances of narrow moral progress that reflect, e.g., improvements in our moral capacities? Most ink has been spilled over the idea of an *expansion of the moral circle*. This is where entities (people or non‐human animals) that were not previously accorded equal moral status, or any moral status at all, come to be seen as having (equal) moral standing. Over time, people come to recognise that factors such as sex, ethnicity, wealth, religion, or species‐membership are not morally relevant categories, so that invoking them cannot be used to justify treating out‐group members differently than in‐group members. This creates a pressure to steadily afford more and more people equal basic moral status, and to afford non‐human animals some form of moral standing (Buchanan & Powell, 2018, 153–158; Singer, 2011). There are several other types of narrow moral progress; what follows is a non‐exhaustive list.

First, moral progress sometimes consists in *proper demoralization*. Homosexuality, sex outside of marriage, masturbation, lending money at interest, and interracial marriage all used to be considered morally wrong, but have now been de‐moralized (Baker, 2019; Buchanan & Powell, 2017; Sauer, 2019). To the extent that these things were incorrectly moralized in the past they were surplus moral constraints that came with large psychological and material costs: the removal of these constraints counts as a form of moral progress.

Relatedly, there are instances of *proper moralization*. This is where behaviours that were previously wrongly seen to be morally neutral come to be seen as having moral status. For instance, workplace sexism used to be considered a morally neutral fact of life, whereas it is now rightly moralized and seen as morally wrong (Buchanan & Powell, 2016, 988; Sauer, 2019, 158).

This example leads neatly to a third type of narrow moral progress, the *improvement of moral concepts*. This can involve the development of entirely new moral concepts, such as the concept of sexual harassment. This concept enables people to understand a certain kind of injustice, and to intelligibly communicate the injustice to others much more effectively than they would be able to if they lacked the concept (Fricker, 2007, 149–152), and so its introduction is a form of moral progress (Buchanan & Powell, 2018, 54). The improvement of moral concepts can also involve the refinement of existing moral concepts. For instance, Buchanan and Powell (2017, 110–111) argue that the concept of responsibility for an action used to put more focus on whether someone had caused a particular outcome and less on whether they had intended to cause the outcome. That our concept of responsibility now places more emphasis on intentionality can be seen as a progressive development of the concept (Buchanan & Powell, 2017, 110–111).

Finally, there are *improvements in moral motivation*. This happens when moral motivation comes to play a more important role in determining people's behaviour, which often leads to greater levels of compliance with moral norms (Buchanan & Powell, 2018, 54–55). Improvements in moral motivation can be seen as a form of narrow moral progress, but often also have implications for forms of broad moral progress. For example, the more people follow norms of their own accord, the less states or communities need to make use of extrinsic motivators in the form of incentives, rewards, and punishments to maintain compliance (Heath, 2014, 177; 2020, 6, 9). Particularly in light of the fact that such punishments often involve brutal “eye‐for‐an‐eye” type treatment (Kitcher, 2011, 140; Pinker, 2011, 144–153; Roth, 2014), to the extent that more effective kinds of socialisation can be pioneered that give people stronger internal motivation to follow moral norms this looks like progress from the point of view of promoting people's welfare.

## 4 CRITERIA FOR MORAL PROGRESS

If we claim that there can be moral progress, then this entails that we can *evaluate* at least some changes in terms of morally better and worse: “claims to the effect that someone or something has (or has not) made moral progress are evaluative terms about certain kinds of changes” (Rønnow‐Rasmussen, 2017: 137; see also Macklin, 1977: 371). What criteria do we or should we use to carry out these kinds of evaluations?

Several authors sidestep this question, likely because it is clearly very difficult or they believe it cannot be answered in a general way. Instead, they start with examples of changes that they assume most people would agree are instances of moral progress, and then aim for theories that can explain how this progress happened (see e.g., Buchanan & Powell, 2018; Sauer, 2019). This research does not aim to make explicit or discuss our criteria for judging that one state of the world is morally better or worse than another state. However, the strategy is not without its critics (e.g., Buddeberg, 2019; Sterelny, 2019), who object that this strategy uncritically presupposes and perhaps even reinforces the status quo, as well as Eurocentric, hegemonic and neo‐colonial ideas.

Of the thinkers who do engage with the question of what the criteria for morally evaluating change are, some argue that moral progress can be measured according to a single criterion. Two examples of this are the amount of overall happiness (an utilitarian criterion), and the proportion of actions in a society that are made with the motive of respecting other people as ends in themselves and not only as means (a deontological criterion; Musschenga & Meynen, 2017).

The most influential contemporary proponent of a single criterion theory of moral progress is the utilitarian Peter Singer. Singer (1993: 12–14; 2011: 96–124) argues that whether a change is progressive depends on whether it expands our circle of moral concern. Consequently, Western societies have progressed morally because their circle of moral concern has come to include more and more people: from single families and tribes, to nations, families of nations (EU), and even all of humanity (Universal Human Rights Declaration). However, Singer (1990; 1993: 242–255), and many others along with him, have been arguing that this is not enough and that we also need to include most non‐human animals and future generations. Some go further still and argue that even nature (woods, rivers, and areas of land) needs to be included in our circle of moral concern (e.g., Stone, 2010).

Other moral philosophers reject the idea that a single criterion can adequately capture something as complex as moral progress, and instead propose pluralistic measures. One such thinker is Martha Nussbaum (2011) with her work on the capabilities approach. She has formulated a list of ten central capabilities that governments as a minimum ought to secure for their citizens, for humans everywhere to be able to have an adequate quality of life. A capability is a freedom to choose what a person is able to do and be in a central area of life. These include “life”, “bodily health”, “bodily integrity”, “senses, imagination, and thought”, “practical reason”, “affiliation”, “other species”, “play” and “control over one's environment” (Nussbaum, 2011: 17–45). The overall measuring rod for moral progress on both an individual, a societal and a cross‐cultural level is such an irreducibly multi‐dimensional and value‐plural conception of a flourishing human life.

While focussing on a single criterion when evaluating change can be perfectly justifiable and even necessary in some cases (e.g., to conduct accurate empirical research), we believe that a multi‐dimensional conception is the more promising general framework for thinking about moral progress. This is because the latter seems truer to the complexities of moral life and will help us avoid moral blindness by fixating on one aspect of what can be morally important, such as, for example, freedom or preferences.

## 5 THE PROSPECTS OF MORAL PROGRESS

Talking about moral progress only makes sense if some states can be morally better (or worse) than other states. Some authors deny that this is possible. For example, some cultural relativists (roughly, philosophers who think that what is morally right and wrong can only be evaluated with reference to specific cultures) think that in order to compare the morality of different states, there needs to be some global frame of reference. But when it comes to morality, they claim, there is no global frame of reference; we only have competing local frames of reference, each belonging to a different culture. Hence, it does not make sense to talk about moral progress (Park, 2011; Shweder, 2004). A similar point could be made by someone who claims that to meaningfully compare the morality of different states, moral realism has to be true (roughly, the view that there are moral truths in the world, independent of what people think, feel, or do), but who think that moral realism is false.

Most philosophers (sensibly, we think) do not deny that moral progress is possible in principle (cf. Buchanan & Powell, 2019, 288–291). However, there is a more serious challenge to the possibility of moral progress: Even if moral progress is possible in principle, the prospects of ever making or sustaining progress in practice may be (very) slim.

The majority of recent discussions have focused on the challenge of evolutionary conservatism (*evoconservatism*). Evoconservatism targets the feasibility of expanding the moral circle. The challenge goes like this: Human moral psychology evolved in an environment of high levels of competition between small groups of hunter‐gatherers. In this environment, more cooperative groups usually had the competitive edge. Morality was therefore adaptive when it promoted within‐group cooperation. In contrast, it was generally not adaptive for groups to extend cooperation beyond the group; quite the opposite: Often, it would have been adaptive to develop strong anti‐social tendencies toward members of competing groups. As a result, humans are left with a moral psychology that is “hard‐wired” to cooperate with members of the in‐group, but to exclude members of the out‐group from the circle of moral concern. Because of this, expanding the moral circle goes against the evolutionary grain of human moral psychology. It will therefore be very hard, if not impossible to ever achieve this type of moral progress (e.g., Fukuyama, 2002; Haidt, 2012).

Several responses to this challenge have been offered. Buchanan and Powell (Buchanan & Powell, 2015, 2018) argue that it is premised on a mistaken view of human moral psychology. They point to various features of contemporary morality that are more inclusive than what should be possible on the evoconservative story—for example, the abolition of slavery, de‐colonialism, and the human rights and animal rights movements. Buchanan and Powell argue that evoconservatives simply cannot explain these developments. Therefore, their view of moral psychology cannot be the full picture. Instead, Buchanan and Powell think that human moral psychology is flexible enough to allow for expansions of the moral circle in certain favourable environments (like those found in some modern societies).

Others have suggested that even if evoconservatives do not have their facts wrong, there may be ways to get around the limits of our evolved moral psychology. One proposed solution is institutional bypassing (Sauer, 2019; see also Sauer, 2018, 83ff.). The idea is for societies to design their institutions in such a way that they either work around, or even harness people's limited willingness to cooperate to achieve expansions of the moral circle. For example, markets involve people cooperating with and placing trust in complete strangers all around the world, even though many are driven to participate in the market by entirely selfish motives (Anomaly, 2017; Heath, 2014).

A second proposal appeals to biomedical moral enhancement—that is, the use of biomedical technology to improve ourselves (our moral motivation, beliefs, moral reasoning, etc.) morally (Douglas, 2008). For example, there is evidence to suggest that the hormone oxytocin increases people's willingness to trust others, and leads to more prosocial behaviour (Persson & Savulescu, 2012). Hence, oxytocin‐based interventions (and interventions like them) could be used to help overcome some of the evolved limitations of our moral psychology that hinder expansions of the moral circle (Persson & Savulescu, 2019).

Discussions of the prospects of moral progress have mostly focussed on expansions of the moral circle; other types of moral progress (last section) have largely been ignored. While the prospects of expanding the moral circle clearly are important, we think that other types of moral progress deserve more attention. Each type of moral progress likely faces its own unique challenges but may also offer unique opportunities (Sauer, 2019). To give just one example, many think that emotions play an important role in what behaviours and beliefs fall under the umbrella of morality (e.g., Haidt, 2001; Prinz, 2007). If this is right, then the decoupling of certain behaviours or beliefs from emotion is likely going to be a key mechanism of proper demoralization. Depending on how easily this can be done, this means that proper demoralization may be much easier (or much harder) to achieve than expansions of the moral circle.

There is other room for improvement, too: literature on the limits of moral progress tends to focus on the limits of our evolved psychology. However, some have made the case it is impossible to properly understand how societies change over time without taking into account cultural evolution (e.g., Henrich, 2016, 2020; Richerson & Boyd, 2006). There may, then, be a set of challenges to the prospects of moral progress quite different from the limitations of our moral psychology. For example, on Henrich's (2016, 2020) account, cultural evolution is such a strong force that when it comes to things like kayaks, languages, family structures and social institutions, “individual people don't design things; the process of cultural evolution designs things” (Kelly & Hoburg, 2017: 10). If this is right, then it may often prove be close to impossible for individuals within a society to steer that society towards moral progress—clearly, this would not be great news for the prospects of intentionally bringing about moral progress (at least progress in the narrow sense, cf. Sect. 1).

## 6 THE METAETHICS OF MORAL PROGRESS

What are the metaethical implications of moral progress? One metaethical question is whether moral progress can be given a fully *naturalistic* account. Most theorists of moral progress believe that a viable explanation of moral change should not upset broadly naturalistic premises (Buchanan & Powell, 2018). It should not be assumed, for instance, that the moral trajectory of history is guided by a supernatural agent. But it is not entirely clear whether a thoroughly naturalistic account of progress is possible, because any explanation of moral progress may at some point have to invoke normative facts (FitzPatrick, 2019).

Another set of central issues arises at the intersection of *epistemology* and moral progress. On one hand, many debates in moral epistemology have straight‐forward implications for the question of how we can know (or if we can ever know) whether moral progress has occurred. To give just one example, if moral skepticism is true, then the answer to the question if we can ever know whether moral progress has occurred may well be “No”: If we cannot have any moral knowledge—because moral judgments are not beliefs, or because no moral judgments are ever true or justified—then we cannot know whether an episode of moral change amounts to progress.

On the other hand, the existence of moral progress may pose *specific* problems for moral epistemology. It is possible, for instance, that moral progress supports moral skepticism via *pessimistic meta‐induction*: if there is moral progress, then we have been deeply wrong about morality before, so how can we rule out that we are not deeply wrong *now* (Stokes, 2017; Williams, 2015)?

However, it is fair to say that the dominant metaethical debate surrounding the issue of moral progress is the connection between moral progress and *moral realism*. One question is whether moral realism *can* explain moral progress. The other is whether objective moral truths *do*, in fact, explain moral change. Some argue that the fact that something is morally wrong can sometimes play a role in explaining its decline or disappearance: various injustices, for instance, lead to social instability (Railton, 1986), unjust social structures are less sustainable (Cohen, 1997). The seemingly more parsimonious explanation that progressive change is caused not by the moral facts, but by people's moral beliefs and attitudes, appears to be incomplete because in many cases, progressive values and beliefs are adopted only *after* certain changes are already underway (Luco, 2019).

Some go further and argue that not only can mind‐independent moral facts explain moral progress, but they are an important part of the best explanation for why moral progress occurs the way it does. Over the past decades and centuries, we have witnessed a precipitous decline in various morally objectionable practices and attitudes such as torture, murder, rape, war, slavery, capital punishment, or colonialism (Pinker, 2011; 2018). Huemer (2016) has argued that non‐realist accounts can neither explain the *recency* nor the *coherent direction* of these changes. Instead, Huemer suggests that these liberal moral shifts are best explained by a rejection of normatively unjustified biases which spread through society in virtue of the fact that the least biased individuals in society also tend to be the most influential because cognitive ability is a common cause of both low bias and high prestige. (A similar story allegedly does not hold for political changes in the direction of increased redistribution and government regulation, Huemer, 2019).

Many remain unconvinced by Huemer's argument. Some have taken issue with the suggestion that non‐realist accounts cannot explain these liberal moral shifts, pointing out that there are a number of alternative explanations which do just this without relying on objective moral facts. For example, functionalist explanations of moral progress hold that moral improvements can be understood in terms of whether moral norms and values become better at discharging their function of overcoming altruism failures (Kitcher, 2011). Descriptively, this idea has some merit, since there is evidence that moral innovations do in fact sometimes solve socially disruptive problems (Curry, 2016; Tomasello, 2016). Others model moral progress after scientific progress, where one framework can be judged as better than another not because it more accurately grasps external truths, but because it enjoys various theoretical advantages (Wilson, 2010).

Others have gone further and taken up Huemer's challenge head on, aiming to provide a non‐realist explanation of moral progress that is *better* than the realist one (Cofnas, 2019; Hopster, 2020). According to these authors, the global shift towards liberal morality is best explained by cultural processes that do not track the moral truth: moral outlooks that respect robust rights, treat people as individuals with equal dignity and discourage violence are culturally attractive because of the safety and protection they provide. More liberal societies also tend to be better at securing cooperation, which makes them less punitive and wealthier. The trend towards liberalism can thus be explained, and better be explained, without assuming the existence of mind‐independent moral facts.

## 7 CONCLUSION

The taboo against the concept of moral progress has been lifted and there is now a lively debate on the topic. If we follow the thinking of Moody‐Adams, then this should be welcomed: “The idea of moral progress is a necessary presupposition of action for beings like us. We must believe that moral progress is possible and that it might have been realized in human experience, if we are to be confident that continued human action can have any morally constructive point” (Moody‐Adams, 2017, 153).

## References

[phc312769-bib-0001] Anomaly, J. (2017). Trust, trade, and moral progress: How market exchange promotes trustworthiness. Social Philosophy and Policy, 34(2), 89–107.

[phc312769-bib-0002] Anscombe, G. E. M. (1958). Modern moral philosophy. Philosophy, 33(124), 1–19.

[phc312769-bib-0003] Baker, R. (2019). The structure of moral revolutions: Studies of changes in the morality of abortion, death, and the bioethics revolution. Cambridge, MA: MIT Press.

[phc312769-bib-0004] Brandhorst, M. (2015). Correspondence to reality in ethics. Philosophical Investigations, 38(3), 227–250.

[phc312769-bib-0005] Buchanan, A. (2020). Our moral fate: Evolution and the escape from tribalism. Cambridge, MA: MIT Press.

[phc312769-bib-0006] Buchanan, A. , & Powell, R. (2015). The limits of evolutionary explanations of morality and their implications for moral progress. Ethics, 126(1), 37–67.

[phc312769-bib-0007] Buchanan, A. , & Powell, R. (2016). Toward a naturalistic theory of moral progress. Ethics, 126(4), 983–1014.

[phc312769-bib-0008] Buchanan, A. , & Powell, R. (2017). De‐moralization as emancipation: Liberty, progress, and the evolution of invalid moral norms. Social Philosophy and Policy, 34(2), 108–135.

[phc312769-bib-0009] Buchanan, A. , & Powell, R. (2018). The evolution of moral progress: A biocultural theory. Oxford: Oxford University Press.

[phc312769-bib-0010] Buchanan, A. , & Powell, R. (2019). Reply to comments. Analyse & Kritik, 41(2), 287–300.

[phc312769-bib-0011] Buddeberg, E. (2019). Is there moral progress? Analyse & Kritik, 41(2), 195–204. 10.1515/auk-2019-0012

[phc312769-bib-0012] Cofnas, N. (2019). A debunking explanation for moral progress. Philosophical Studies. Advance online publication.

[phc312769-bib-0013] Cohen, J. (1997). The arc of the moral universe. Philosophy & Public Affairs, 26(2), 91–134.

[phc312769-bib-0014] Curry, O. S. (2016). Morality as cooperation: A problem‐centred approach. In T. K. Shackelford & R. D. Hansen (Eds.), The evolution of morality (pp. 27–51). Springer International Publishing.

[phc312769-bib-0015] Douglas, T. (2008). Moral enhancement. Journal of Applied Philosophy, 25(3), 228–245. 19132138 10.1111/j.1468-5930.2008.00412.x PMC2614680

[phc312769-bib-0016] Eriksen, C. (2020). Moral change: Dynamics, structure and normativity. New York: Palgrave Macmillan.

[phc312769-bib-0017] FitzPatrick, W. J. (2019). Moral progress for evolved rational creatures. Analyse & Kritik, 41(2), 217–238.

[phc312769-bib-0018] Fricker, M. (2007). Epistemic injustice: Power and the ethics of knowing. Oxford: Oxford University Press.

[phc312769-bib-0019] Fukuyama, F. (2002). Our posthuman future: Consequences of the biotechnology revolution. Farrar, Straus and Giroux.

[phc312769-bib-0020] Haidt, J. (2001). The emotional dog and its rational tail: A social intuitionist approach to moral judgment. Psychological Review, 108(4), 814–834. 11699120 10.1037/0033-295x.108.4.814

[phc312769-bib-0021] Haidt, J. (2012). The righteous mind: Why good people are divided by politics and religion. Vintage.

[phc312769-bib-0022] Heath, J. (2014). Morality, competition, and the firm: The market failures approach to business ethics. Oxford: Oxford University Press.

[phc312769-bib-0023] Heath, J. (2020). Is Socialism Atavistic?. In D. J. D'Amico & A. G. Martin (Eds.), Philosophy, Politics, and Austrian Economics, Advances in Austrian Economics (Vol. 25, pp. 49–65). Bingley: Emerald Publishing Limited. 10.1108/S1529-213420200000025003

[phc312769-bib-0024] Henrich, J. (2016). The secret of our success: How culture is driving human evolution, domesticating our species, and making us smarter. Princeton University Press.

[phc312769-bib-0025] Henrich, J. (2020). The weirdest people in the world: How the West became psychologically peculiar and particularly prosperous. Farrar, Straus and Giroux.

[phc312769-bib-0026] Hermann, J. (2019). The dynamics of moral progress. Ratio, 32(4), 300–311.

[phc312769-bib-0027] Hopster, J. (2020). Explaining historical moral convergence: The empirical case against realist intuitionism. Philosophical Studies, 177(5), 1255–1273.

[phc312769-bib-0028] Huemer, M. (2016). A liberal realist answer to debunking skeptics: The empirical case for realism. Philosophical Studies, 173(7), 1983–2010.

[phc312769-bib-0029] Huemer, M. (2019). Debunking leftward progress. Ratio, 32(4), 312–324.

[phc312769-bib-0030] Kelly, D. , & Hoburg, P. (2017). A tale of two processes: On Joseph Henrich's the secret of our success: How culture is driving human evolution, domesticating our species, and making us smarter. Philosophical Psychology, 30(6), 832–848.

[phc312769-bib-0031] Kitcher, P. (2011). The ethical project. Harvard University Press.

[phc312769-bib-0032] Kitcher, P. (2017). Social progress. Social Philosophy and Policy, 34(2), 46–65. 10.1017/S0265052517000206

[phc312769-bib-0033] Luco, A. (2019). How moral facts cause moral progress. Journal of the American Philosophical Association, 5(4), 429–448.

[phc312769-bib-0034] MacIntyre, A. (1981). After virtue. University of Notre Dame Press.

[phc312769-bib-0035] Macklin, R. (1977). Moral progress. Ethics, 87(4), 370–382.

[phc312769-bib-0036] Moody‐Adams, M. M. (1999). The idea of moral progress. Metaphilosophy, 30(3), 168–183.

[phc312769-bib-0037] Moody‐Adams, M. M. (2017). Moral progress and human agency. Ethical Theory & Moral Practice, 20(1), 153–168.

[phc312769-bib-0038] Musschenga, A. W. , & Meynen, G. (2017). Moral progress: An introduction. Ethical Theory & Moral Practice, 20(1), 3–15.

[phc312769-bib-0039] Nussbaum, M. C. (1993). Non‐relative virtues: An Aristotelian approach. In M. C. Nussbaum & A. Sen (Eds.), The quality of life (pp. 242–269). Oxford: Oxford University Press.

[phc312769-bib-0040] Nussbaum, M. C. (2011). Creating capabilities: The human development approach. The Belknap Press of Harvard University Press.

[phc312769-bib-0041] Park, S. (2011). Defence of cultural relativism. La Cultura, 8(1), 159–170.

[phc312769-bib-0042] Persson, I. , & Savulescu, J. (2012). Unfit for the future? Human nature, scientific progress, and the need for moral enhancement. In G. Kahane , J. Savulescu , & R. T. Meulen (Eds.), Enhancing human capacities (pp. 486–500). Oxford: Oxford University Press.

[phc312769-bib-0043] Persson, I. , & Savulescu, J. (2019). The evolution of moral progress and biomedical moral enhancement. Bioethics, 33(7), 814–819. 31107561 10.1111/bioe.12592 PMC6766868

[phc312769-bib-0044] Pinker, S. (2011). The better angels of our nature: A history of violence and humanity. Penguin Books.

[phc312769-bib-0045] Pinker, S. (2018). Enlightenment now: The case for reason, science, humanism and progress. Penguin Books.

[phc312769-bib-0046] Posner, R. A. (1997a). The problematics of moral and legal theory. Harvard Law Review, 111(7), 1637–1717.

[phc312769-bib-0047] Posner, R. A. (1997b). Reply to critics of the problematics of moral and legal theory. Harvard Law Review, 111(7), 1796–1823.

[phc312769-bib-0048] Prinz, J. (2007). The emotional construction of morals. Oxford: Oxford University Press.

[phc312769-bib-0049] Railton, P. (1986). Moral realism. Philosophical Review, 95(2), 163–207.

[phc312769-bib-0050] Richerson, P. J. , & Boyd, R. (2006). Not by genes alone: How culture transformed human evolution. University of Chicago Press.

[phc312769-bib-0051] Rønnow‐Rasmussen, T. (2017). On locating value in making moral progress. Ethical Theory & Moral Practice, 20(1), 137–152.

[phc312769-bib-0052] Roser, M. , Ortiz‐Ospina, E. , & Ritchie, H. (2013). Life expectancy. Our World in Data. https://ourworldindata.org/life-expectancy

[phc312769-bib-0053] Roth, M. P. (2014). An eye for an eye: A global history of crime and punishment. Reaktion Books Ltd.

[phc312769-bib-0054] Sauer, H. (2018). Moral thinking, fast and slow. Routledge.

[phc312769-bib-0055] Sauer, H. (2019). Butchering benevolence moral progress beyond the expanding circle. Ethical Theory & Moral Practice, 22(1), 153–167.

[phc312769-bib-0056] Shweder, R. A. (2004). George W. Bush & the missionary position. Dædalus, 133(3), 26–36.

[phc312769-bib-0057] Singer, P. (1990). Animal liberation. Avon Books (Original work published 1975).

[phc312769-bib-0058] Singer, P. (1993). Practical ethics. Cambridge: Cambridge University Press. 39946524

[phc312769-bib-0059] Singer, P. (2011). The expanding circle–Ethics, evolution, and moral progress. Princeton University Press (Original work published 1981).

[phc312769-bib-0060] Sterelny, K. (2019). Evolutionary foundations for a theory of moral progress? Analyse & Kritik, 41(2), 205–216. 10.1515/auk-2019-0013

[phc312769-bib-0061] Stokes, P. (2017). Towards a new epistemology of moral progress. European Journal of Philosophy, 25(4), 1824–1843.

[phc312769-bib-0062] Stone, C. D. (2010). Should trees have standings?–Law, morality and the environment. Oxford University Press.

[phc312769-bib-0063] Tomasello, M. (2016). A natural history of human morality. Harvard University Press.

[phc312769-bib-0064] Williams, B. (1985). Ethics and the limits of philosophy. Harvard University Press.

[phc312769-bib-0065] Williams, E. G. (2015). The possibility of an ongoing moral catastrophe. Ethical Theory & Moral Practice, 18(5), 971–982.

[phc312769-bib-0066] Wilson, C. (2010). Moral progress without moral realism. Philosophical Papers, 39(1), 97–116.

